# Tricuspid regurgitation: a hidden risk factor for atrial fibrillation related stroke?

**DOI:** 10.3389/fcvm.2023.1135069

**Published:** 2023-07-18

**Authors:** Yong Soo Kim, Han-Gil Jeong, In-Chang Hwang, Beom Joon Kim, Joon-Myung Kwon, Hee-Joon Bae, Moon-Ku Han

**Affiliations:** ^1^ Department of Neurology, Seoul National University Bundang Hospital, Seoul National University College of Medicine, Seongnam, Republic of Korea; ^2^ Department of Neurology, Nowon Eulji Medical Center, Eulji University School of Medicine, Seoul, Republic of Korea; ^3^ Division of Neurocritical Care, Department Of Neurosurgery and Neurology, Seoul National University Bundang Hospital, Seoul National University College of Medicine, Seongnam, Republic of Korea; ^4^ Division of Cardiology, Department of Internal Medicine, Seoul National University Bundang Hospital, Seoul National University College of Medicine, Seongnam, Republic of Korea; ^5^ Department of Critical Care and Emergency Medicine, Mediplex Sejong Hospital, Incheon, Republic of Korea

**Keywords:** atrial fibrillation, atrial cardiopathy, ischemic stroke, stroke subtype, tricuspid regurgitation

## Abstract

**Background and purpose:**

Tricuspid regurgitation (TR) is a common but overlooked valvular disease, and its association with the etiologic subtypes of ischemic stroke is unclear. We explored the relationship between TR and atrial fibrillation (AF) in patients with acute ischemic stroke.

**Methods:**

This retrospective analysis of ongoing stroke registry assessed 6,886 consecutive acute ischemic stroke patients who underwent transthoracic echocardiography during their in-hospital care. Multivariable logistic regression models adjusted for age, sex, stroke characteristics, and echocardiographic indices were used to investigate the association between TR and total AF, and newly diagnosed AF during hospitalization and a 1-year follow-up period, respectively.

**Results:**

TR was present in 877 (12.7%) patients (mild, 9.9%; moderate, 2.4%; severe, 0.5%). AF was identified in 24.1% (medical history, 11.1%; first detected in the emergency room, 6.6%; newly diagnosed after admission, 6.4%). TR was associated with AF [adjusted odds ratio (aOR) 4.87 (95% confidence interval (CI), 2.63–9.03)], compared with no/trivial TR. The association between TR and AF was consistent regardless of severity (aOR [95% CI], 4.57 [2.63–7.94] for mild and 7.05 [2.57–19.31] for moderate-to-severe TR) or subtype of TR (5.44 [2.91–10.14] for isolated and 3.81 [2.00–7.28] for non-isolated TR). Among the AF-naïve patients at admission, TR was associated with newly diagnosed AF during hospitalization and a 1-year follow-up period (aOR [95% CI], 2.68 [1.81–3.97]).

**Conclusions:**

TR is associated with AF in acute ischemic stroke patients regardless of severity and subtypes of TR. TR is also associated with newly diagnosed AF after stroke.

## Introduction

More than 20% of ischemic stroke cases are caused by cardiac thromboembolism resulting from to atrial fibrillation (AF), and the prevalence of AF among stroke patients is increasing (1). AF-related strokes are more fatal or disabling compared to strokes caused by other etiologies and have the highest in-hospital mortality among ischemic stroke (2, 3). Because most AF-related strokes can be prevented by oral anticoagulants (OAC), echocardiographic or electrophysiologic biomarkers of AF have been studied to detect subclinical AF for effective prevention of stroke. However, previous predictors of hidden AF, including left atrial (LA) enlargement, have primarily focused on the left heart (4). Considering recent evidence linking right atrial (RA) structural remodeling to the development of incident AF, biomarkers associated with right heart may play a supportive role in estimating the risk of subclinical AF (5).

Tricuspid regurgitation (TR), a common valvular heart disease but long considered the “forgotten valve”, is one condition that may be associated with right heart remodeling. Over 90% of TR cases are functional and occurs secondary to various left-sided heart diseases or pulmonary hypertension (6, 7). Long-standing AF is another significant risk factor for TR, as it causes remodeling of the RA and tricuspid valve annulus (8). Moderate or severe TR occurs in approximately 30% of patients with newly diagnosed AF, and rhythm control of AF reduces the risk of TR (9). However, although it has recently been suggested that atrial cardiopathy, which can exist without overt AF, can potentially promote the development of AF and cause ischemic stroke, whether TR related right heart remodeling is a precursor to AF is not well defined (4, 10). Right-sided heart disease induces re-entrant activity in the RA through RA remodeling and can create a substrate for AF maintenance (11). Thus, TR could potentially serve as a surrogate marker or an accelerating factor of AF by causing atrial remodeling.

Understanding the association between TR and subclinical AF can help in the selection of appropriate candidates for extensive cardiac monitoring to detect AF in patients with ischemic stroke. In this study, our aim was to investigate the relationship between TR characteristics and AF in patients with acute ischemic stroke and determine whether TR is associated with newly diagnosed AF in AF-naïve stroke patients.

## Methods

### Patient selection

The 11,623 consecutive patients with stroke admitted to a single tertiary referral hospital between January 1, 2011 and December 31, 2020 were screened for this observational, retrospective, case–control study. We included individuals who had ischemic stroke or lesion-positive transient ischemic attack (TIA) confirmed by neuroimaging (*n* = 9,409), and further selected those who underwent transthoracic echocardiography (TTE) during their in-hospital care for acute stroke (*n* = 6,901). Lesion positive TIA was defined when the stroke symptom improved within 24 h and there was no evidence of stroke on initial brain imaging, but the ischemic lesion was identified on follow-up brain imaging. After excluding 15 patients without information on tricuspid valve function on TTE, 6,886 patients were finally included in this analysis.

This study involving human participants was reviewed and approved by the institutional review board of Seoul National University Bundang Hospital (approval number: B-2108/701-102). The requirement for written informed consent was waived by the institutional review board of Seoul National University Bundang Hospital due to the retrospective nature of the study.

### Clinical data collection

Clinical information of patients, including demographics, vascular risk factors, and stroke characteristics were obtained from our prospectively collected stroke registry database. Data on the following vascular risk factors were retrieved for the study: history of stroke, hypertension, diabetes mellitus, hyperlipidemia, AF, smoking habits, and anemia. The initial severity of stroke upon arrival at the emergency department was measured using the National Institutes of Health Stroke Scale (NIHSS) score. Functional status before and after the stroke event was rated using the modified Rankin Scale (mRS).

AF was defined when a patient or family member self-reported that the patient had been diagnosed with AF prior to the stroke event, or when AF was diagnosed by 12-lead electrocardiogram (ECG), Holter monitoring, or implantable cardiac monitoring. When AF was suspected during the continuous ECG monitoring in a stroke unit or an intensive care unit, examination by 12-lead ECG was required to confirm AF diagnosis. AF-naïve patients were those with no diagnosis of AF at the time of admission by medical history or by the first 12-lead ECG in the emergency department. Paroxysmal AF was defined when normal sinus rhythm was restored within 7 days, while sustained AF was defined when the AF lasted more than 7 days (12). To investigate the presence of new onset AF in AF-naïve patients, the findings from 12-lead ECG, Holter monitoring, and implantable cardiac monitoring devices during hospitalization and over a 1-year follow-up period were reviewed.

The extent of diagnostic evaluation and acute treatment strategies were determined according to the institutional protocols, based on the guidelines of the American Heart Association/American Stroke Association, and were at the discretion of the stroke management physician (13).

### Echocardiography indices and tricuspid regurgitation

TTE images obtained during admission were investigated. Echocardiographic assessments were performed according to the current guidelines of the American Society of Echocardiography and included M-mode, two-dimensional, and Doppler echocardiographic measurements (14). Mitral *E* velocity, septal mitral annular e′ velocity, E/e′ ratio, left ventricular (LV) ejection fraction, LA volume index, right ventricular (RV) systolic pressure, LV end-diastolic volume, LV end-systolic volume, LV mass index, severity of TR, aortic stenosis (AS), aortic regurgitation (AR), mitral stenosis (MS), and mitral regurgitation (MR) were assessed. Severity of TR was graded according to the structural findings (i.e., RA/RV size and TV morphology) of tricuspid valve, qualitative Doppler assessment, and semi-quantitative assessment. Regarding trivial TR as insignificant and due to small number of patients with severe TR, the severity of TR was divided into three categories: no or trivial TR, mild TR, and moderate-to-severe TR.

Isolated TR was diagnosed when the following conditions were met: (1) TR holosystolic and functional, (2) absence of pulmonary hypertension (RV systolic pressure <50 mmHg), (3) LV ejection fraction ≥50%, (4) absence of other moderate or severe valvular heart diseases, (5) absence of pacemaker or defibrillator wire across the tricuspid valve, (6) absence of congenital or pericardial disease, or (7) no history of valve surgery (15). Alternatively, TR was classified as non-isolated TR.

### Statistical analysis

We compared the clinical and echocardiographic characteristics of patients according to the presence of TR and AF, using *χ*^2^ tests for categorical variables and *t*-tests for continuous variables, respectively. Multivariable logistic regression models were constructed to test the association of AF in acute ischemic stroke patients with the presence, severity, and subtype of TR. As a subgroup analysis, the association between TR and newly diagnosed AF during hospitalization and during the 1-year follow-up was assessed in patients who were AF-naïve at the time of admission. Adjustments for confounders were performed for variables with bivariate *p*-values <0.10 or for those that were considered clinically relevant. Multiple-imputation chained equations were used to impute the missing echocardiographic indices (16). The results of the regression models were reported using odds ratios (ORs) with 95% confidence interval (CIs), as appropriate.

Details of the multivariable models, data imputation, and complete case analysis are provided in Supplementary Methods. The levels of statistical significance were set at a *p*-value of 0.05 for two-tailed tests. The statistical analyses were performed using R version 4.0.3 (R Development Core Team, Vienna, Austria). The R packages used in this analysis were “MASS”, “Hmisc”, “tidyverse”, “descr”, “readxl”, “compareGroups”, “car” and “mice”.

## Results

### Baseline characteristics

Among the 11,623 patients who were screened for the study, 6,886 patients were finally eligible for this retrospective analysis (Supplementary Figure S1). A comparison of the eligible patients with the excluded patients (without TTE) is presented in Supplementary Table S1. The mean [SD] age of the eligible patients was 67.9 [13.4] years; 4,103 [59.6%] were male, and 1,409 [20.5%] had a history of stroke or TIA. Among the eligible patients, 6,859 [99.6%] were admitted to a stroke unit or an intensive care unit for a median duration of 20 h [interquartile range (IQR) 18–48 h]. At least one 12-lead ECG measurement was obtained in 99.0% of patients [2 (1–4) times per patient]. Holter and implantable cardiac monitoring were performed in 3,791 [55.1%] and 107 [1.6%] of the patients, respectively. The median [IQR] time from admission to TTE examination was 2.5 [1.4–4.3] days. AF was diagnosed in 1,658 [24.1%] of the patients, 1,216 [73.3%] of whom were diagnosed by medical history or first 12-lead ECG at the emergency department, while 442 [26.7%] were newly diagnosed during hospitalization or during the 1-year follow-up. Paroxysmal AF and sustained AF were detected in 518 [31.2%] and 1,140 [68.8%] of patients, respectively.

### Patient characteristics according to TR

In the population with ischemic stroke, TR (≥mild) was observed in 877 [12.7%] of the patients, and the severity of TR was mild in 680 [9.9%], moderate in 164 [2.4%], and severe in 33 [0.5%]. Isolated TR and non-isolated TR was present in 498 [7.2%] and 364 [5.3%] of the patients, respectively. TR was due to primary tricuspid abnormalities in 3 patients, pacemaker/defibrillator *in situ* in 23 patients, abnormal right ventricular dilatation in 34 patients, previous valve surgery in 42 patients, and suspected congenital heart disease in 31 patients. Patients with TR were older and were more often dependent on others prior to stroke onset (mRS ≥ 3), had more severe neurological symptoms at onset, and had more multifocal acute ischemic lesions on cerebral imaging. Patients with TR more often had a history of stroke and hypertension, and less frequently had diabetes mellitus or smoked, compared with those without TR. AF was more frequently present in patients with TR than in patients without TR (594 [67.7%] vs. 1,064 [17.7%], *p*-value <0.01). Patients with TR had a lower LV ejection fraction, higher E/e′ ratio, higher LA volume index, and more often presented with left-sided valvular heart disease than their counterpart (Table 1). A comparison of the patient's characteristics according to the severity and subtype of TR is provided in Supplementary Table S2.

**Table 1:** Patient characteristics according to the presence of tricuspid regurgitation.

	No/trivial TR (*n* = 6,009)	≥Mild TR (*n* = 877)	*p*-Value
Baseline characteristics			
Age, years	66.7 ± 13.5	75.8 ± 10.1	<0.01
Male sex, *n* (%)	3,734 (62.1)	369 (42.1)	<0.01
Premorbid dependency (mRS ≥ 3), *n* (%)	409 (6.8)	110 (12.5)	<0.01
Baseline NIHSS score, median (IQR)	3 (1–7)	5 (2–15)	<0.01
Affected vascular territory			
Anterior circulation, *n* (%)	3,641 (60.6)	603 (68.8)	<0.01
Posterior circulation, *n* (%)	1,995 (33.2)	203 (23.1)	
Both, *n* (%)	373 (6.2)	71 (8.1)	
Infarct location			
Deep^a^, *n* (%)	2,376 (39.5)	213 (24.3)	<0.01
Cortical^b^, *n* (%)	2,851 (47.4)	513 (58.5)	
Both, *n* (%)	782 (13.0)	151 (17.2)	
Stroke classification			
Large-artery atherosclerosis, *n* (%)	2,507 (41.7)	153 (17.4)	<0.01
Small-vessel occlusion, *n* (%)	1,009 (16.8)	48 (5.5)	
Cardioembolism, *n* (%)	1,176 (19.6)	541 (61.7)	
Other determined etiology, *n* (%)	371 (6.2)	22 (2.5)	
Undetermined etiology, *n* (%)	946 (15.7)	113 (12.9)	
Reperfusion therapy			
Intravenous thrombolysis, *n* (%)	582 (9.7)	154 (17.6)	<0.01
Endovascular treatment, *n* (%)	739 (12.3)	174 (19.8)	<0.01
Stroke risk factors			
Previous stroke, *n* (%)	1,193 (19.9)	216 (24.6)	<0.01
Hypertension, *n* (%)	4,180 (69.6)	659 (75.1)	<0.01
Diabetes mellitus, *n* (%)	2,019 (33.6)	220 (25.1)	<0.01
Hyperlipidemia, *n* (%)	2,089 (34.8)	301 (34.3)	0.83
Smoking, *n* (%)	2,541 (42.3)	226 (25.8)	<0.01
Atrial fibrillation, *n* (%)	1,064 (17.7)	594 (67.7)	<0.01
Previously diagnosed, *n* (%)	421 (7.0)	342 (39.0)	<0.01
First detected in the emergency room, *n* (%)	293 (4.9)	160 (18.2)	
Newly diagnosed after admission, *n* (%)	350 (5.8)	92 (10.5)	
Paroxysmal, *n* (%)	402 (6.7)	116 (13.2)	<0.01
Sustained, *n* (%)	662 (11.0)	478 (54.5)	
Echocardiographic parameters			
LV ejection fraction, %	61.8 ± 7.7	59.0 ± 9.6	<0.01
LV end-diastolic volume, ml	75.0 ± 24.8	69.3 ± 26.9	<0.01
LV end-systolic volume, ml	29.4 ± 15.9	29.6 ± 18.3	0.78
LV mass index, g/m^2^	97.9 ± 25.8	105.0 ± 29.1	<0.01
LA volume index, ml/m^2^	37.4 ± 16.1	63.6 ± 32.4	<0.01
Mitral E/e′	11.5 ± 5.3	15.6 ± 9.3	<0.01
RV systolic pressure, mmHg	26.4 ± 5.9	37.1 ± 10.2	<0.01
AS ≥ moderate, *n* (%)	42 (0.7)	16 (1.8)	<0.01
AR ≥ moderate, *n* (%)	50 (0.8)	24 (2.7)	<0.01
MS ≥ moderate, *n* (%)	16 (0.3)	24 (2.7)	<0.01
MR ≥ moderate, *n* (%)	39 (0.7)	69 (7.9)	<0.01
Outcome			
Poor functional outcome at 3 months (mRS ≥ 3), *n* (%)	1,869 (31.1)	413 (47.1)	<0.01
Mortality at 3 months, *n* (%)	271 (4.5)	67 (7.6)	<0.01
Vascular death	75 (1.2%)	17 (2.0%)	0.07
Other death	196 (3.3%)	50 (5.7%)	<0.01
Mortality at 1 year, *n* (%)	490 (8.2)	136 (15.5)	<0.01
Vascular death	99 (1.7%)	27 (3.1%)	<0.01
Other death	391 (6.5%)	109 (12.4%)	<0.01

Values are mean ± standard deviation, median (interquartile range), or frequency (%). TR, tricuspid regurgitation; mRS, modified Rankin Scale; NIHSS, National Institute of Health Stroke Scale; LV, left ventricle; LA, left atrium; RV, right ventricle; AS, aortic stenosis; AR, aortic regurgitation; MS, mitral stenosis; MR, mitral regurgitation.

^a^ Deep location refers to basal ganglia, thalamus, internal capsule, corona radiata, and brainstem.

^b^ Cortical location refers to cerebral cortex and cerebellum.

### Association between TR and AF in patients with stroke

**Figure 1:**
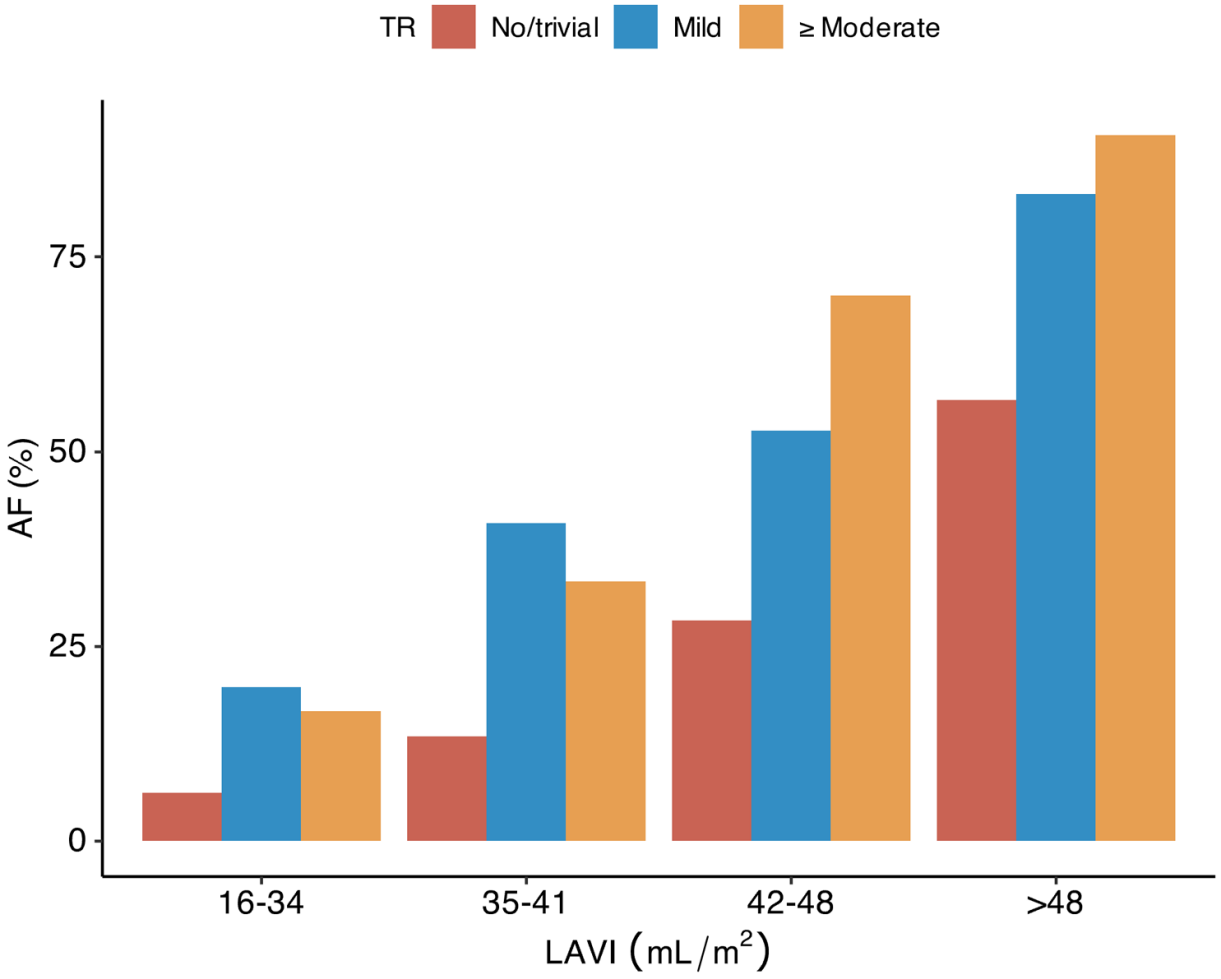
Tricuspid regurgitation, LA volume index, and atrial fibrillation in patients with acute ischemic stroke. TR, tricuspid regurgitation; LA, left atrium; LAVI, left atrial volume index; AF, atrial fibrillation.

Patients with AF were older than those without AF and their neurological deficit at baseline was more severe (Supplementary Table S3). According to TTE, patients with AF had a lower LV ejection fraction, a higher E/e′ ratio, a higher LA volume index, a higher RV systolic pressure, and more left-sided valvular heart disease. TR was observed in 594 [35.8%] of patients with AF (mild TR, 434 [26.2%]; moderate-to-severe TR, 160 [9.7%]) and in 283 [5.4%] of patients without AF (mild TR, 246 [4.7%]; moderate-to-severe TR, 37 [0.7%]). Isolated TR accounted for 315 [19.4%] of the patients with AF and 183 [3.6%] of the patients without AF. The prevalence of AF increased with increasing severity of TR, in all strata of the LA volume index (Figure 1).

The multivariable logistic regression model showed a significant association between the presence of any type of TR and AF (adjusted OR [aOR], 4.87 [95% CI 2.63–9.03]) (Table 2). Mild TR [aOR, 4.57 (2.63–7.94)], as well as moderate-to-severe TR [aOR 7.05 (2.57–19.31)], was associated with AF compared to no/trivial TR. Both isolated TR [aOR 5.44 (2.91–10.14)] and non-isolated TR [aOR 3.81 (2.00–7.28)] showed a significant association with AF. Details of the multivariable models are described in Supplementary Table S4. Complete case analyses without multiple imputations showed similar results (Supplementary Table S5).

**Table 2:** Multivariable logistic regression analyses for atrial fibrillation in patients with acute ischemic stroke.

		Crude OR (95% CI)	*p*-Value	Adjusted OR (95% CI)	*p*-Value
Any TR		9.75 (8.34–11.41)	<0.01	4.87 (2.63–9.03)	<0.01
TR severity	No/trivial TR	Reference		Reference	
	Mild TR	8.20 (6.92–9.72)	<0.01	4.57 (2.63–7.94)	<0.01
	≥Moderate TR	20.10 (13.97–28.91)	<0.01	7.05 (2.57–19.31)	<0.01
TR subtype	No/trivial TR	Reference		Reference	
	Isolated TR	8.03 (6.62–9.75)	<0.01	5.44 (2.91–10.14)	<0.01
	Non-isolated TR	12.94 (10.18–16.45)	<0.01	3.81 (2.00–7.28)	<0.01

Multivariable model was adjusted for age, sex, baseline NIHSS, premorbid mRS, infarct location, previous stroke, hypertension, smoking, anemia, E/e′ ratio, LV ejection fraction, RV systolic pressure, LV end-diastolic volume, LV mass index, LA volume index, aortic regurgitation ≥ moderate, aortic stenosis ≥ moderate, mitral regurgitation ≥ moderate, and mitral stenosis ≥ moderate.

OR, odds ratio; CI, confidence interval; TR, tricuspid regurgitation; NIHSS, National Institutes of Health Stroke Scale; mRS, modified Rankin Scale; LV, left ventricle; RV, right ventricle; LA, left atrium.

### Newly diagnosed AF after admission

In subgroup of 5,576 AF-naïve patients at the time of admission, AF was newly diagnosed in 442 [7.9%] patients during admission or during the 1-year follow-up (Table 3): by 12-lead ECG in 330 [5.9%], by Holter monitoring in 86 [1.5%], and by an implantable cardiac monitoring device in 26 [0.5%]. The detection rate of newly diagnosed AF was higher in patients with TR than in patients without TR, in all strata of the LA volume index (Supplementary Figure S2). The presence of TR was associated with the detection of AF during admission and during the 1-year follow-up [aOR 2.68 (1.81–3.97)] (Table 4). In addition, in patients without AF at discharge, the presence of TR was also associated with newly diagnosed AF after discharge (Supplementary Table S6).

**Table 3:** Comparison of patients according to newly diagnosed atrial fibrillation after ischemic stroke in AF-naïve patients at admission.

	No AF (*n* = 5,134)	Newly diagnosed AF (*n* = 442)	*p*-Value
Baseline characteristics			
Age, years	66.0 ± 13.8	72.9 ± 10.7	<0.01
Male sex, *n* (%)	3,128 (60.9)	245 (55.4)	0.03
Premorbid dependency (mRS ≥ 3), *n* (%)	336 (6.5)	47 (10.6)	<0.01
Baseline NIHSS score, median (IQR)	3 (1–6)	5 (2–15)	<0.01
Affected vascular territory			
Anterior circulation, *n* (%)	3,036 (59.1)	296 (67.1)	<0.01
Posterior circulation, *n* (%)	1,779 (34.7)	107 (24.2)	
Both, *n* (%)	319 (6.2)	39 (8.8)	
Infarct location			
Deep^a^, *n* (%)	2,260 (44.0)	86 (19.5)	<0.01
Cortical^b^, *n* (%)	2,258 (44.0)	280 (63.3)	
Both, *n* (%)	616 (12.0)	76 (17.2)	
Stroke classification			
Large-artery atherosclerosis, *n* (%)	2,533 (49.3)	69 (15.6)	<0.01
Small-vessel occlusion, *n* (%)	1,019 (19.8)	12 (2.7)	
Cardioembolism, *n* (%)	389 (7.6)	269 (61.0)	
Other determined etiology, *n* (%)	371 (7.2)	16 (3.6)	
Undetermined etiology, *n* (%)	822 (16.0)	76 (17.2)	
Intravenous thrombolysis, *n* (%)	398 (7.8)	84 (19.0)	<0.01
Endovascular treatment, *n* (%)	516 (10.1)	91 (20.6)	<0.01
Reperfusion therapy			
Intravenous thrombolysis			
Endovascular therapy			
Stroke risk factors			
Previous stroke, *n* (%)	975 (19.0)	98 (22.2)	0.12
Hypertension, *n* (%)	3,502 (68.2)	310 (70.1)	0.44
Diabetes mellitus, *n* (%)	1,702 (33.2)	134 (30.3)	0.24
Hyperlipidemia, *n* (%)	1,766 (34.4)	158 (35.7)	0.60
Smoking, *n* (%)	2,207 (43.0)	143 (32.4)	<0.01
Echocardiographic parameters			
LV ejection fraction, %	62.4 ± 7.2	60.1 ± 9.7	<0.01
LV end-diastolic volume, ml	75.3 ± 24.1	71.8 ± 28.0	0.01
LV end-systolic volume, ml	29.0 ± 14.9	29.9 ± 20.4	0.37
LV mass index, g/m^2^	97.1 ± 25.5	101.0 ± 28.5	0.02
LA volume index, ml/m^2^	34.3 ± 11.1	46.0 ± 18.2	<0.01
Mitral E/e′	11.2 ± 5.0	13.2 ± 7.2	<0.01
RV systolic pressure, mmHg	26.6 ± 6.5	29.9 ± 9.0	<0.01
Any TR, *n* (%)	268 (5.2)	92 (20.8)	<0.01
Mild TR, *n* (%)	234 (4.6)	76 (17.2)	<0.01
Moderate TR, *n* (%)	29 (0.6)	16 (3.6)	
Severe TR, *n* (%)	5 (0.1)	0 (0)	
Isolated TR, *n* (%)	177 (3.5)	52 (12.0)	<0.01
Non-isolated TR, *n* (%)	86 (1.7)	40 (9.3)	
AS ≥ moderate, *n* (%)	32 (0.6)	4 (0.9)	0.53
AR ≥ moderate, *n* (%)	47 (0.9)	6 (1.4)	0.31
MS ≥ moderate, *n* (%)	7 (0.1)	5 (1.1)	<0.01
MR ≥ moderate, *n* (%)	27 (0.5)	12 (2.7)	<0.01

Values are mean ± standard deviation, median (interquartile range), or frequency (%). AF, atrial fibrillation; mRS, Modified Rankin Scale; NIHSS, National institute of health stroke scale; LV, left ventricle; LA, left atrium; RV, right ventricle; TR, tricuspid regurgitation; AS, aortic stenosis; AR, aortic regurgitation; MS, mitral stenosis; MR, mitral regurgitation.

^a^ Deep location refers to basal ganglia, thalamus, internal capsule, corona radiata and brainstem.

^b^ Cortical location refers to cerebral cortex and cerebellum.

**Table 4:** Multivariable logistic regression analyses for newly diagnosed atrial fibrillation after ischemic stroke in AF-naïve patients at admission.

		Crude OR (95% CI)	*p*-Value	Adjusted OR (95% CI)	*p*-Value
Any TR		4.77 (3.68–6.19)	<0.01	2.68 (1.81–3.97)	<0.01
TR severity	No/trivial TR	Reference		Reference	
	Mild TR	4.52 (3.41–5.98)	<0.01	2.68 (1.81–3.97)	<0.01
	≥Moderate TR	6.54 (3.58–11.97)	<0.01	2.64 (1.19–5.90)	0.02
TR subtype	No/trivial TR	Reference		Reference	
	Isolated TR	4.05 (2.91–5.62)	<0.01	2.94 (1.89–4.57)	<0.01
	Non-isolated TR	6.22 (4.22–9.18)	<0.01	2.19 (1.28–3.76)	<0.01

Multivariable model was adjusted for age, sex, baseline NIHSS, premorbid mRS, infarct location, previous stroke, hypertension, smoking, anemia, E/e′ ratio, LV ejection fraction, RV systolic pressure, LV end-diastolic volume, LV mass index, LA volume index, aortic regurgitation ≥ moderate, aortic stenosis ≥ moderate, mitral regurgitation ≥ moderate, and mitral stenosis ≥ moderate.

OR, odds ratio; CI, confidence interval; TR, tricuspid regurgitation; NIHSS, National Institutes of Health Stroke Scale; mRS, modified Rankin Scale; LV, left ventricle; RV, right ventricle.

## Discussion

In our study, TR was significantly associated with AF in patients with acute ischemic stroke, and the association was evident even in those with mild TR and isolated functional TR. These results imply that TR may play a significant role in increasing susceptibility to AF. Newly diagnosed AF during hospitalization and the follow-up period was more frequently observed in patients with TR than in patients without TR. This finding suggests that TR needs to be considered as a potential risk factor for subclinical AF in the stroke population.

Cardiac evaluation in patients with ischemic stroke has focused on the left side of the heart because the thrombus causing cardioembolic stroke is mostly formed in the left heart chamber. However, as the heart is a single organ that has a connected tissue structure and electrical conduction system, both sides of the heart can interact in promoting atrial remodeling, which affects the initiation, maintenance, and progression of AF. Recently, an additional role of right-sided heart disease on the initiation or maintenance of AF has been attracting interest. Pulmonary artery hypertension or chronic obstructive lung disease are known to be related to AF (11, 17, 18). The resulting right heart remodeling can promote right atrial remodeling, which can act as a vulnerable intracardiac substrate for AF (11, 19). Our study investigated the association between TR, as a surrogate marker of right atrial remodeling, and AF in patients with acute ischemic stroke.

Chronic AF causes bilateral atrial enlargement (20). The LA volume index has previously been shown to have a positive relationship with AF in stroke patients, but the cut-off value of the LA volume index for defining a population with a high risk of AF is unclear (4, 21). AF-related dilatation of the atrium results in atrioventricular valve annulus dilation and enhances retrograde flow during systole (22). Due to the relatively thin right atrial wall and less developed fibrous skeleton of the annulus, the tricuspid valve is more prone to AF-related cardiac structural remodeling than the mitral valve (22–24). This phenomenon was also observed in this study, as AF was more frequently accompanied by TR than by MR, which implies that right atrial remodeling, reflected as TR, may be useful for identifying patients with a high risk of AF.

Previously, TR was considered as a consequence of tricuspid valve annulus dilatation due to long-standing AF-associated RA remodeling (6). However, in this study, approximately 24% of AF cases detected in patients with TR were paroxysmal. This suggests that TR may also serve to initiate and maintain paroxysmal AF that is sufficient to cause cardioembolic stroke. AF is typically thought to originate in the triggering foci located at the pulmonary vein myocardial sleeve (25). However, pulmonary vein isolation with catheter ablation was less effective in persistent AF cases, and several non-pulmonary vein triggers, such as those in the RA and coronary sinus, were suggested as additional ablation targets (26). The RA is one of the candidates for an AF substrate, but the factors causing RA remodeling remain unclear (27–30). TR may be a surrogate marker of RA remodeling that directly causes RA-related AF or secondary changes in LA-related AF, which can further aggravate RA remodeling and AF through a vicious cycle in patients with ischemic stroke.

In approximately 20% of patients with ischemic stroke, no probable cause of stroke can be identified after adequate diagnostic evaluation (31, 32). Subclinical AF accounts for 10%–15% of these undetermined causes of stroke, and identifying subclinical AF is crucial for appropriate secondary prevention of stroke (33, 34). Even if the index stroke was a non-AF related stroke, such as stroke due to large artery atherosclerosis or small vessel disease, AF detection after stroke usually compels physicians to initiate anticoagulation therapy. Atrial cardiopathy or atrial conduction abnormality including Bayes syndrome without overt AF is a high-risk cause of embolism in patients with an undetermined cause of stroke, and long-term monitoring of cardiac rhythm in this population is reasonable (10, 35, 36). The efficacy of anticoagulants in patients with evidence of atrial cardiopathy and cryptogenic stroke is being investigated in a randomized multicenter clinical trial (37). The association of TR and newly diagnosed AF in our study suggests that patients with TR may benefit from long-term cardiac monitoring to detect subclinical AF.

Moderate-to-severe TR, traditionally known as a bystander of left-sided heart disease, has recently been acknowledged to be associated with poor cardiovascular outcome (38). Although mild TR has been considered a benign echocardiographic finding due to its limited hemodynamic effect, recent reports have demonstrated that mild TR could progress and lead to cardiovascular morbidities, such as new onset AF (39). The association between mild TR and AF in our study suggests the potential role of mild TR in thromboembolic events and as a marker or promoter of atrial cardiopathy and AF.

This study had several limitations. First, this was a retrospective, single-center study with wide period of enrollment, implying that selection bias might have existed. Approximately 27% of patients with ischemic stroke or lesion positive TIA were excluded because an echocardiogram was not performed. Second, we did not have quantified measurements of right heart remodeling, including RA diameter or volume, RV systolic function, RV dimension, or TR severity at the time of AF detection. Additionally, unmeasured confounders, including the presence of thyroid disease, may have affected the relationship between TR and AF. Thus, this study does not provide sufficient evidence of a causal relationship between TR and AF, which is still speculative. Third, advanced cardiac images to assess tricuspid valve structure, including cardiac MRI, was not evaluated, which may underestimate the organic causes of TR. Fourth, the intensity of diagnostic work-up for AF was very heterogenous and all data from continuous ECG monitoring in the stroke units or intensive care units were not systemically reviewed. Fifth, we did not investigate the prevalence and risk of ischemic stroke in the general population with TR. Sixth, since we considered trivial TR as non-significant finding, hemodynamic effects of trivial TR may be underestimated. Lastly, we used multiple imputations, and the possible discrepancy between imputed data and real data should be considered.

In conclusion, our study found that TR was significantly associated with AF in patients with stroke, even in cases of mild or isolated TR. Furthermore, TR was associated with newly diagnosed AF during admission and during the 1-year follow-up period after ischemic stroke. TR, due to atrial remodeling or as a promoter of atrial cardiopathy, may be closely associated with the occurrence of cardioembolic stroke attributable to AF. Future studies investigating the causal relationship between preexisting TR and the development of new AF will help understanding atrial cardiopathy, which may be a potential treatment target in patients with ischemic stroke. Presence of significant TR and right heart dysfunction needs to be considered in future studies aiming to find hidden AF in patients with stroke.

## Data Availability

The raw data supporting the conclusions of this article will be made available by the authors, upon request, to any qualified researcher.
