# Supplementary material for: Tricuspid regurgitation: a hidden risk factor for atrial fibrillation related stroke?
Source: Front Cardiovasc Med. 2023 Jul 18;10:1135069. doi: 10.3389/fcvm.2023.1135069 (PMC10400321; doi:10.3389/fcvm.2023.1135069)
Supplement: Supplementary file 1 [file Table1.docx]

***Supplementary material***

**Supplementary Methods**

To investigate the association between tricuspid regurgitation (TR) and atrial fibrillation (AF) in patients with stroke, multivariable logistic regression models were constructed. In our dataset, there were missing values in several echocardiographic indices: E/e’ ratio (108 [1.6%]), LV ejection fraction (117 [1.7%]), LV end-diastolic volume (121 [1.8%]), LV end-systolic volume (121 [1.8%]), LV mass index (45 [0.7%]), and LA volume index (566 [8.2%]). We employed multiple imputation chained equations to impute the missing variables and generated five imputed datasets.

As the sensitivity analyses, bivariate analysis, univariable and multivariable logistic regression analysis were conducted, using complete cases without missing values (n=6205). In this analysis, patients with any missing data (681 [9.9%] patients) were excluded.

**Supplementary Table 1. Comparison of the Eligible Patients and Patients Excluded Due to Lack of Transthoracic Echocardiographic Evaluation**

|  | **Eligible Patients (n=6886)** | **No TTE  (n=2523)** | ***P-*value** |
| --- | --- | --- | --- |
| **Baseline characteristics** |  |  |  |
| Age, years | 67.9 ± 13.4 | 67.3 ± 14.0 | 0.07 |
| Male sex, n (%) | 4103 (59.6) | 1412 (56.0) | <0.01 |
| Premorbid dependency (mRS ≥ 3), n (%) | 519 (7.5) | 243 (9.6) | <0.01 |
| Baseline NIHSS score, median (IQR) | 3 (1–8) | 3 (1–7) | <0.01 |
| Affected vascular territory |  |  |  |
| Anterior circulation, n (%) | 4244 (61.7) | 1525 (61.5) | 0.54 |
| Posterior circulation, n (%) | 2196 (31.9) | 810 (32.6) |  |
| Both, n (%) | 444 (6.5) | 146 (5.9) |  |
| Infarct location |  |  |  |
| Deep^a^, n (%) | 2589 (37.6) | 1119 (44.4) | <0.01 |
| Cortical^b^, n (%) | 3364 (48.9) | 1088 (43.1) |  |
| Both, n (%) | 933 (13.5) | 316 (12.5) |  |
| Stroke classification |  |  |  |
| Large-artery atherosclerosis, n (%) | 2660 (38.6) | 1061 (42.7) | <0.01 |
| Small-vessel occlusion, n (%) | 1059 (15.4) | 505 (20.3) |  |
| Cardioembolism, n (%) | 1715 (24.9) | 419 (16.9) |  |
| Other determined etiology, n (%) | 393 (5.7) | 251 (10.1) |  |
| Undetermined etiology, n (%) | 1059 (15.4) | 248 (10.0) |  |
| Intravenous thrombolysis, n (%) | 736 (10.7) | 184 (7.3) | <0.01 |
| Endovascular treatment, n (%) | 913 (13.3) | 237 (9.4) | <0.01 |
| **Stroke risk factors** |  |  |  |
| Previous stroke, n (%) | 1409 (20.5) | 761 (30.2) | <0.01 |
| Hypertension, n (%) | 4839 (70.3) | 1746 (69.2) | 0.33 |
| Diabetes mellitus, n (%) | 2239 (32.5) | 865 (34.3) | 0.11 |
| Hyperlipidemia, n (%) | 2390 (34.7) | 1035 (41.0) | <0.01 |
| Smoking, n (%) | 2767 (40.2) | 959 (38.0) | 0.06 |
| Atrial fibrillation, n (%) | 1658 (24.1) | 402 (15.9) | <0.01 |
| **Functional outcome** |  |  |  |
| Poor functional outcome at 3 months (mRS ≥ 3), n (%) | 2282 (33.1) | 804 (31.9) | 0.25 |
| Mortality at 3 months, n (%) | 338 (4.9) | 200 (7.9) | <0.01 |

^a^Deep location refers to basal ganglia, thalamus, internal capsule, corona radiata and brainstem; ^b^Cortical location refers to cerebral cortex and cerebellum. TTE, transthoracic echocardiography; mRS, modified Rankin Scale; NIHSS, National Institute of Health Stroke Scale.

**Supplementary Table 2. Patient Characteristics According to The Severity and Subtypes of Tricuspid Regurgitation**

|  | **No/trivial TR (n=6009)** | **TR Severity** | | **TR Subtype** | | ***P-*value**^a^ | ***P-*value**^b^ |
| --- | --- | --- | --- | --- | --- | --- | --- |
|  |  | **Mild  (n=680)** | ≥ **Moderate (n=197)** | **Isolated (n=498)** | **Non-isolated (n=364)** |  |  |
| **Baseline characteristics** |  |  |  |  |  |  |  |
| Age, years | 66.7 ± 13.5 | 75.2 ± 10.4 | 77.9 ± 9.0 | 75.8 ± 9.6 | 75.9 ± 10.8 | <0.01 | <0.01 |
| Male sex, n (%) | 3734 (62.1) | 302 (44.4) | 67 (34.0) | 212 (42.6) | 148 (40.7) | <0.01 | <0.01 |
| Premorbid dependency (mRS ≥ 3), n (%) | 409 (6.8) | 84 (12.4) | 26 (13.2) | 55 (11.0) | 52 (14.3) | <0.01 | <0.01 |
| Baseline NIHSS score, median (IQR) | 3 (1 –7) | 5 (2–14) | 7 (2–17) | 5 (2–13) | 7 (2–16) | <0.01 | <0.01 |
| Affected vascular territory |  |  |  |  |  |  |  |
| Anterior circulation, n (%) | 3641 (60.6) | 457 (67.2) | 146 (74.1) | 348 (69.9) | 248 (68.1) | <0.01 | <0.01 |
| Posterior circulation, n (%) | 1995 (33.2) | 167 (24.6) | 36 (18.3) | 119 (23.9) | 78 (21.4) |  |  |
| Both, n (%) | 373 (6.2) | 56 (8.2) | 15 (7.6) | 31 (6.2) | 38 (10.4) |  |  |
| Infarct location |  |  |  |  |  |  |  |
| Deep^c^, n (%) | 2376 (39.5) | 167 (24.6) | 46 (23.4) | 138 (27.7) | 71 (19.5) | <0.01 | <0.01 |
| Cortical^d^, n (%) | 2851 (47.4) | 401 (59.0) | 112 (56.9) | 282 (56.6) | 223 (61.3) |  |  |
| Both, n (%) | 782 (13.0) | 112 (16.5) | 39 (19.8) | 78 (15.7) | 70 (19.2) |  |  |
| Stroke classification |  |  |  |  |  |  |  |
| Large-artery atherosclerosis, n (%) | 2507 (41.7) | 136 (20.0) | 17 (8.6) | 106 (21.3) | 43 (11.8) | <0.01 | <0.01 |
| Small-vessel occlusion, n (%) | 1011 (16.8) | 44 (6.5) | 4 (2.0) | 37 (7.4) | 11 (3.0) |  |  |
| Cardioembolism, n (%) | 1174 (19.5) | 390 (57.4) | 151 (76.6) | 263 (52.8) | 268 (73.6) |  |  |
| Other determined etiology, n (%) | 371 (6.2) | 19 (2.8) | 3 (1.5) | 10 (2.0) | 11 (3.0) |  |  |
| Undetermined etiology, n (%) | 946 (15.7) | 91 (13.4) | 22 (11.2) | 82 (16.5) | 31 (8.5) |  |  |
| Intravenous thrombolysis, n (%) | 582 (9.7) | 114 (16.8) | 40 (20.3) | 81 (16.3) | 68 (18.7) | <0.01 | <0.01 |
| Endovascular treatment, n (%) | 739 (12.3) | 123 (18.1) | 51 (25.9) | 87 (17.5) | 82 (22.5) | <0.01 | <0.01 |
| **Stroke risk factors** |  |  |  |  |  |  |  |
| Previous stroke, n (%) | 1193 (19.9) | 164 (24.1) | 52 (26.4) | 126 (25.3) | 87 (23.9) | <0.01 | 0.10 |
| Hypertension, n (%) | 4180 (69.6) | 504 (74.1) | 155 (78.7) | 386 (77.5) | 262 (72.0) | <0.01 | <0.01 |
| Diabetes mellitus, n (%) | 2019 (33.6) | 181 (26.6) | 39 (19.8) | 121 (24.3) | 94 (25.8) | <0.01 | <0.01 |
| Hyperlipidemia, n (%) | 2089 (34.8) | 232 (34.1) | 69 (35.0) | 160 (32.1) | 136 (37.4) | 0.94 | 0.27 |
| Smoking, n (%) | 2541 (42.3) | 191 (28.1) | 35 (17.8) | 132 (26.5) | 92 (25.3) | <0.01 | <0.01 |
| Atrial fibrillation, n (%) | 1064 (17.7) | 434 (63.8) | 160 (81.2) | 315 (63.3) | 270 (74.2) | <0.01 | <0.01 |
| Previous history of AF, n (%) | 421 (7.0) | 246 (36.2) | 96 (48.7) | 176 (35.3) | 161 (44.2) | <0.01 | <0.01 |
| AF diagnosed at ED, n (%) | 293 (4.9) | 112 (16.5) | 48 (24.4) | 87 (17.5) | 69 (19.0) |  |  |
| AF diagnosed after admission, n (%) | 350 (5.8) | 76 (11.2) | 16 (8.1) | 52 (10.4) | 40 (11.0) |  |  |
| Paroxysmal, n (%) | 402 (6.7) | 102 (15.0) | 14 (7.1) | 61 (12.2) | 54 (14.8) | <0.01 | <0.01 |
| Sustained, n (%) | 662 (11.0) | 332 (48.8) | 146 (74.1) | 254 (51.0) | 216 (59.3) |  |  |
| **Echocardiographic parameters** |  |  |  |  |  |  |  |
| LV ejection fraction, % | 61.8 ± 7.7 | 59.1 ± 9.3 | 58.6 ± 10.4 | 62.3 ± 5.2 | 54.5 ± 12.0 | <0.01 | <0.01 |
| LV end-diastolic volume, mL | 75.0 ± 24.8 | 70.7 ± 27.3 | 64.7 ± 25.0 | 64.7 ± 19.6 | 75.8 ± 33.5 | <0.01 | <0.01 |
| LV end-systolic volume, mL | 29.4 ± 15.9 | 29.9 ± 18.3 | 28.2 ± 18.5 | 24.5 ± 8.5 | 36.6 ± 24.8 | 0.41 | <0.01 |
| LV mass index, g/m^2^ | 97.9 ± 25.8 | 103.0 ± 28.6 | 109.0 ± 30.5 | 99.5 ± 24.7 | 112.0 ± 33.1 | <0.01 | <0.01 |
| LA volume index, mL/m^2^ | 37.4 ± 16.1 | 58.9 ± 25.3 | 79.9 ± 46.0 | 55.1 ± 22.4 | 75.3 ± 39.6 | <0.01 | <0.01 |
| Mitral E/e’ | 11.5 ± 5.3 | 15.3 ± 9.1 | 16.5 ± 9.8 | 12.8 ± 5.0 | 19.6 ± 12.1 | <0.01 | <0.01 |
| RV systolic pressure, mmHg | 26.4 ± 5.9 | 35.7 ± 9.4 | 42.0 ± 11.5 | 34.0 ± 6.9 | 41.5 ± 12.0 | <0.01 | <0.01 |
| AS ≥ moderate, n (%) | 42 (0.7) | 13 (1.9) | 3 (1.5) | 0 (0) | 16 (4.4) | <0.01 | <0.01 |
| AR ≥ moderate, n (%) | 50 (0.8) | 16 (2.4) | 8 (4.1) | 0 (0) | 24 (6.6) | <0.01 | <0.01 |
| MS ≥ moderate, n (%) | 16 (0.3) | 17 (2.5) | 7 (3.6) | 0 (0) | 24 (6.6) | <0.01 | <0.01 |
| MR ≥ moderate, n (%) | 39 (0.7) | 41 (6.0) | 28 (14.2) | 0 (0) | 69 (19.0) | <0.01 | <0.01 |
| **Outcome** |  |  |  |  |  |  |  |
| Poor functional outcome at 3 months (mRS ≥ 3), n (%) | 1869 (31.1) | 305 (44.9) | 108 (54.8) | 211 (42.4) | 192 (52.7) | <0.01 | <0.01 |
| Mortality at 3 months, n (%) | 271 (4.5) | 49 (7.2) | 18 (9.1) | 26 (5.2) | 39 (10.7) | <0.01 | <0.01 |

^a^*P-*values for TR severity; ^b^*P-*values for TR subtype; ^c^Deep location refers to basal ganglia, thalamus, internal capsule, corona radiata and brainstem; ^d^Cortical location refers to cerebral cortex and cerebellum. TR, tricuspid regurgitation; mRS, modified Rankin Scale; NIHSS, National institute of health stroke scale; AF, atrial fibrillation; ED, emergency department; LV, left ventricle; LA, left atrium; RV, right ventricle; AS, aortic stenosis; AR, aortic regurgitation; MS, mitral stenosis; MR, mitral regurgitation.

**Supplementary Table 3. Characteristics According to the Time of Diagnosis of Atrial Fibrillation**

|  | **No AF  (n=5228)** | **Previous History of AF (n=763)** | **AF Diagnosed at ED (n=453)** | **AF Diagnosed after Admission (n=442)** | ***P-*value** |
| --- | --- | --- | --- | --- | --- |
| **Baseline characteristics** |  |  |  |  |  |
| Age, years | 66.1± 13.8 | 74.0 ± 10.0 | 73.6 ± 11.2 | 72.9 ± 10.7 | <0.01 |
| Male sex, n (%) | 3179 (60.8) | 411 (53.9) | 268 (59.2) | 245 (55.4) | <0.01 |
| Premorbid dependency  (mRS ≥ 3), n (%) | 346 (6.6) | 84 (11.0) | 42 (9.3) | 47 (10.6) | <0.01 |
| Baseline NIHSS score, median (IQR) | 3 (1—6) | 7 (2—16) | 8 (3—16) | 5 (2—5) | <0.01 |
| Affected vascular territory |  |  |  |  |  |
| Anterior circulation, n (%) | 3096 (59.2) | 540 (70.8) | 312 (68.9) | 296 (67.1) | <0.01 |
| Posterior circulation, n (%) | 1809 (34.6) | 174 (22.8) | 108 (23.8) | 107 (24.2) |  |
| Both, n (%) | 323 (6.2) | 49 (6.4) | 33 (7.3) | 39 (8.8) |  |
| Infarct location |  |  |  |  |  |
| Deep^a^, n (%) | 2299 (44.0) | 128 (16.8) | 76 (16.8) | 86 (19.5) | <0.01 |
| Cortical^b^, n (%) | 2301 (44.0) | 498 (65.3) | 285 (62.9) | 280 (63.3) |  |
| Both, n (%) | 628 (12.0) | 137 (18.0) | 92 (20.3) | 76 (17.2) |  |
| Stroke classification |  |  |  |  |  |
| Large-artery atherosclerosis, n (%) | 2571 (49.2) | 15 (2.0) | 5 (1.1) | 69 (15.6) | <0.01 |
| Small-vessel occlusion, n (%) | 1039 (19.9) | 6 (0.8) | 2 (0.4) | 12 (2.7) |  |
| Cardioembolism, n (%) | 410 (7.8) | 647 (84.8) | 389 (85.9) | 269 (61.0) |  |
| Other determined etiology, n (%) | 375 (7.2) | 2 (0.3) | 0 (0.0) | 16 (3.6) |  |
| Undetermined etiology, n (%) | 833 (15.9) | 93 (12.2) | 57 (12.6) | 76 (17.2) |  |
| Intravenous thrombolysis, n (%) | 411 (7.86) | 138 (18.1) | 103 (22.7) | 84 (19.0) | 0.65 |
| Endovascular treatment, n (%) | 528 (10.1) | 178 (23.3) | 116 (25.6) | 91 (20.6) | 0.91 |
| **Stroke risk factors** |  |  |  |  |  |
| Previous stroke, n (%) | 991 (19.0) | 250 (32.8) | 70 (15.5) | 98 (22.2) | <0.01 |
| Hypertension, n (%) | 3,569 (68.3) | 605 (79.3) | 355 (78.4) | 310 (70.1) | <0.01 |
| Diabetes mellitus, n (%) | 1727 (33.0) | 242 (31.7) | 136 (30.0) | 134 (30.3) | 0.37 |
| Hyperlipidemia, n (%) | 1796 (34.4) | 322 (42.2) | 114 (25.2) | 158 (35.7) | <0.01 |
| Smoking, n (%) | 2238 (42.8) | 223 (29.2) | 163 (36.0) | 143 (32.4) | <0.01 |
| **Echocardiographic parameters** |  |  |  |  |  |
| LV ejection fraction, % | 62.4 ± 7.2 | 58.4 ± 9.7 | 57.5 ± 9.8 | 60.1 ± 9.7 | <0.01 |
| LV end-diastolic volume, mL | 75.3 ± 24.1 | 69.4 ± 28.3 | 72.3 ± 27.2 | 71.8 ± 28.0 | <0.01 |
| LV end-systolic volume, mL | 29.0 ± 14.9 | 30.2 ± 19.7 | 31.9 ± 19.1 | 29.9 ± 20.4 | <0.01 |
| LV mass index, g/m^2^ | 97.2 ± 25.5 | 106.0 ± 28.4 | 104.0 ± 26.6 | 101.0 ± 28.5 | <0.01 |
| LA volume index, mL/m^2^ | 34.5 ± 11.9 | 68.0 ± 33.0 | 61.4 ± 25.4 | 46.0 ± 18.2 | <0.01 |
| Mitral E/e’ | 11.2 ± 5.0 | 15.3 ± 9.1 | 14.1 ± 7.9 | 13.2 ± 7.2 | <0.01 |
| RV systolic pressure, mmHg | 26.6 ± 6.5 | 32.4 ± 9.7 | 30.6 ± 8.6 | 29.9 ± 9.0 | <0.01 |
| Any TR, n (%) | 283 (5.4) | 342 (44.8) | 160 (35.3) | 92 (20.8) | <0.01 |
| Mild TR, n (%) | 246 (4.7) | 246 (32.2) | 112 (24.7) | 76 (17.2) | <0.01 |
| ≥ Moderate TR, n (%) | 37 (0.7) | 96 (12.6) | 48 (10.6) | 16 (3.6) |  |
| Isolated TR, n (%) | 183 (3.6) | 176 (23.4) | 87 (19.7) | 52 (12.0) | <0.01 |
| Non-isolated TR, n (%) | 94 (1.8) | 161 (21.4) | 69 (15.6) | 40 (9.3) |  |
| AS ≥ moderate, n (%) | 32 (0.6) | 13 (1.7) | 9 (2.0) | 4 (0.9) | <0.01 |
| AR ≥ moderate, n (%) | 47 (0.9) | 13 (1.7) | 8 (1.8) | 6 (1.4) | 0.07 |
| MS ≥ moderate, n (%) | 7 (0.1) | 21 (2.8) | 7 (1.6) | 5 (1.1) | <0.01 |
| MR ≥ moderate, n (%) | 28 (0.5) | 46 (6.0) | 22 (4.9) | 12 (2.7) | <0.01 |
| **Functional outcome** |  |  |  |  |  |
| Poor functional outcome at 3 months (mRS ≥ 3), n (%) | 1,496 (28.6) | 350 (45.9) | 215 (47.5) | 221 (50.0) | <0.01 |
| Mortality at 3 months, n (%) | 207 (4.0) | 52 (6.8) | 33 (7.3) | 46 (10.4) | <0.01 |

^a^Deep location refers to basal ganglia, thalamus, internal capsule, corona radiata and brainstem; ^b^Cortical location refers to cerebral cortex and cerebellum. AF, atrial fibrillation; ED, emergency department; mRS, modified Rankin Scale; NIHSS, National Institute of Health Stroke Scale; LV, left ventricle; LA, left atrium; RV, right ventricle; TR, tricuspid regurgitation; AS, aortic stenosis; AR, aortic regurgitation; MS, mitral stenosis; MR, mitral regurgitation.

**Supplementary Table 4. Details of Multivariable Logistic Regression Analyses for Atrial Fibrillation in Patients with Acute Ischemic Stroke**

|  | **Adjusted OR (95% CI)** | ***P-*value** |
| --- | --- | --- |
| **Any TR** |  |  |
| TR | 4.87 (2.63–9.03) | <0.01 |
| Age, per 1 year increase | 1.02 (1.004–1.03) | 0.02 |
| Male sex | 1.86 (1.21–2.86) | 0.01 |
| Premorbid mRS ≥ 3 | 0.51 (0.38–0.67) | <0.01 |
| Baseline NIHSS score, per 1 point increase | 1.07 (1.06–1.09) | <0.01 |
| Vascular territory: anterior | Reference | - |
| Vascular territory: posterior | 0.999 (0.84–1.19) | 0.99 |
| Vascular territory: both | 2.63 (2.07–3.35) | <0.01 |
| Infarct location: deep | Reference | - |
| Infarct location: cortical | 3.10 (2.59–3.71) | <0.01 |
| Infarct location: both | 2.63 (2.07–3.35) | <0.01 |
| Previous stroke | 1.09 (0.92–1.30) | 0.30 |
| Hypertension | 1.13 (0.94 –1.36) | 0.18 |
| Smoking | 0.73 (0.61–0.86) | <0.01 |
| Anemia | 1.74 (1.38–2.18) | <0.01 |
| Mitral E/e’ ratio | 1.01 (0.97–1.04) | 0.71 |
| LV ejection fraction, per 1 increase | 0.97 (0.93–1.001) | 0.053 |
| LV end-diastolic volume, per 1 mL increase | 0.99 (0.97–1.001) | 0.06 |
| LV mass index, per 1 g/m^2^ increase | 1.002 (0.99–1.01) | 0.65 |
| LA volume index, per 1 mL/m^2^ increase | 1.03 (0.94–1.12) | 0.40 |
| RV systolic pressure, per 1 mmHg increase | 1.01 (0.99–1.02) | 0.29 |
| AS ≥ moderate | 1.07 (0.44–2.58) | 0.87 |
| AR ≥ moderate | 1.05 (0.54–2.04) | 0.88 |
| MS ≥ moderate | 2.75 (0.60–12.57) | 0.18 |
| MR ≥ moderate | 1.74 (0.75–4.04) | 0.18 |
| **TR severity** |  |  |
| No/trivial TR | Reference | - |
| Mild TR | 4.57 (2.63–7.94) | <0.01 |
| ≥ Moderate TR | 7.05 (2.57–19.31) | <0.01 |
| Age, per 1 year increase | 1.02 (1.004–1.03) | 0.02 |
| Male sex | 1.86 (1.21–2.86) | 0.01 |
| Premorbid mRS ≥ 3 | 0.51 (0.38–0.67) | <0.01 |
| Baseline NIHSS score, per 1 point increase | 1.08 (1.06–1.09) | <0.01 |
| Vascular territory: anterior | Reference | - |
| Vascular territory: posterior | 1.002 (0.84–1.19) | 0.98 |
| Vascular territory: both | 0.76 (0.57–1.02) | 0.07 |
| Infarct location: deep | Reference | - |
| Infarct location: cortical | 3.11 (2.60–3.73) | <0.01 |
| Infarct location: both | 2.63 (2.07–3.35) | <0.01 |
| Previous stroke | 1.09 (0.92–1.30) | 0.30 |
| Hypertension | 1.13 (0.94–1.36) | 0.18 |
| Smoking | 0.73 (0.62–0.86) | <0.01 |
| Anemia | 1.75 (1.39–2.20) | <0.01 |
| Mitral E/e’ ratio | 1.01 (0.97–1.04) | 0.70 |
| LV ejection fraction, per 1 increase | 0.97 (0.93–1.001) | 0.052 |
| LV end-diastolic volume, per 1 mL increase | 0.99 (0.97–1.001) | 0.052 |
| LV mass index, per 1 g/m^2^ increase | 1.002 (0.99–1.01) | 0.65 |
| LA volume index, per 1 mL/m^2^ increase | 1.03 (0.94–1.12) | 0.41 |
| RV systolic pressure, per 1 mmHg increase | 1.01 (0.99–1.02) | 0.35 |
| AS ≥ moderate | 1.08 (0.45–2.63) | 0.85 |
| AR ≥ moderate | 1.05 (0.54–2.05) | 0.88 |
| MS ≥ moderate | 2.73 (0.60–12.54) | 0.18 |
| MR ≥ moderate | 1.70 (0.74–3.91) | 0.20 |
| **TR subtype** |  |  |
| No/trivial TR | Reference | - |
| Isolated TR | 5.44 (2.91–10.14) | <0.01 |
| Non-isolated TR | 3.81 (2.00–7.28) | <0.01 |
| Age, per 1 year increase | 1.02 (1.004–1.03) | 0.02 |
| Male sex | 1.87 (1.22–2.88) | 0.01 |
| Premorbid mRS ≥ 3 | 0.51 (0.39–0.67) | <0.01 |
| Baseline NIHSS score, per 1 point increase | 1.07 (1.06–1.09) | <0.01 |
| Vascular territory: anterior | Reference |  |
| Vascular territory: posterior | 1.001 (0.84–1.19) | 0.99 |
| Vascular territory: both | 0.77 (0.57–1.02) | 0.07 |
| Infarct location: deep | Reference |  |
| Infarct location: cortical | 3.11 (2.60–3.72) | <0.01 |
| Infarct location: both | 2.64 (2.08–3.36) | <0.01 |
| Previous stroke | 1.09 (0.92–1.29) | 0.31 |
| Hypertension | 1.13 (0.94–1.36) | 0.19 |
| Smoking | 0.73 (0.61–0.86) | <0.01 |
| Anemia | 1.73 (1.38–2.18) | <0.01 |
| Mitral E/e’ ratio | 1.01 (0.97–1.04) | 0.67 |
| LV ejection fraction, per 1 increase | 0.97 (0.93–0.999) | 0.049 |
| LV end-diastolic volume, per 1 mL increase | 0.99 (0.97–1.001) | 0.058 |
| LV mass index, per 1 g/m^2^ increase | 1.002 (0.99–1.01) | 0.65 |
| LA volume index, per 1 mL/m^2^ increase | 1.03 (0.94–1.12) | 0.41 |
| RV systolic pressure, per 1 mmHg increase | 1.01 (0.99–1.02) | 0.20 |
| AS ≥ moderate | 1.12 (0.47–2.69) | 0.79 |
| AR ≥ moderate | 1.11 (0.58–2.16) | 0.75 |
| MS ≥ moderate | 2.85 (0.62–13.10) | 0.17 |
| MR ≥ moderate | 1.92 (0.83–4.43) | 0.12 |

OR, odds ratio; CI, confidence interval; TR, tricuspid regurgitation; mRS, modified Rankin Scale; NIHSS, National Institutes of Health Stroke Scale; LV, left ventricle; LA, left atrium; RV, right ventricle; AS, aortic stenosis; AR, aortic regurgitation; MS, mitral stenosis; MR, mitral regurgitation.

**Supplementary Table 5. Multivariable Logistic Regression Analyses for Diagnosing AF in Acute Ischemic Stroke Patients: Complete Cases (n=6,205)**

|  | | **Crude OR (95% CI)** | ***P-*value** | **Adjusted OR (95% CI)** | ***P-*value** |
| --- | --- | --- | --- | --- | --- |
| **Any TR** | | 10.34 (8.77–12.19) | <0.01 | 3.36 (2.62–4.32) | <0.01 |
| **TR severity** | No/trivial TR | Reference |  | Reference |  |
|  | Mild TR | 8.55 (7.17–10.23) | <0.01 | 3.30 (2.54–4.28) | <0.01 |
|  | ≥ Moderate TR | 23.44 (15.98–35.54) | <0.01 | 3.90 (2.24–6.96) | <0.01 |
| **TR subtype** | No/trivial TR | Reference |  | Reference |  |
|  | Isolated TR | 8.43 (6.90–10.32) | <0.01 | 3.79 (2.86–5.03) | <0.01 |
|  | Non-isolated TR | 14.12 (10.97–18.34) | <0.01 | 2.49 (1.66–3.75) | <0.01 |

Multivariable model was adjusted for age, sex, baseline NIHSS, premorbid mRS, infarct location, previous stroke, hypertension, smoking, anemia, E/e’ ratio, LV ejection fraction, RV systolic pressure, LV end-diastolic volume, LV mass index, LA volume index, aortic regurgitation ≥ moderate, aortic stenosis ≥ moderate, mitral regurgitation ≥ moderate, and mitral stenosis ≥ moderate
AF, atrial fibrillation; OR, odds ratio; CI, confidence interval; TR, tricuspid regurgitation; NIHSS, National Institutes of Health Stroke Scale; mRS, modified Rankin Scale; LV, left ventricle; RV, right ventricle; LA, left atrium.

**Supplementary Table 6. Multivariable Logistic Regression Analyses for Newly Diagnosed Atrial Fibrillation in AF-naïve patients at discharge, additionally adjusted for stroke subtype**

|  | | **Crude OR (95% CI)** | ***P*-value** | **Adjusted OR (95% CI)** | ***P*-value** |
| --- | --- | --- | --- | --- | --- |
| **Any TR** | | 3.75 (2.40–5.87) | <0.01 | 2.33 (1.32–4.11) | <0.01 |
| **TR severity** | No/trivial TR | Reference |  | Reference |  |
|  | Mild TR | 3.95 (2.48–6.29) | <0.01 | 2.68 (1.52–4.74) | <0.01 |
|  | ≥ Moderate TR | 2.37 (0.56–9.96) | 0.24 | 0.64 (0.12–3.40) | 0.60 |
| **TR subtype**^a^ | No/trivial TR | Reference |  | Reference |  |
|  | Isolated TR | 3.16 (1.78–5.61) | <0.01 | 2.73 (1.42–5.23) | <0.01 |
|  | Non-isolated TR | 4.92 (2.56–9.44) | <0.01 | 1.76 (0.73–4.20) | 0.21 |

Multivariable model was adjusted for age, sex, baseline NIHSS, premorbid mRS, infarct location, previous stroke, hypertension, smoking, anemia, E/e’ ratio, LV ejection fraction, RV systolic pressure, LV end-diastolic volume, LV mass index, LA volume index, aortic regurgitation ≥ moderate, aortic stenosis ≥ moderate, mitral regurgitation ≥ moderate, and mitral stenosis ≥ moderate
AF, atrial fibrillation; OR, odds ratio; CI, confidence interval; TR, tricuspid regurgitation; NIHSS, National Institutes of Health Stroke Scale; mRS, modified Rankin Scale; LV, left ventricle; RV, right ventricle; LA, left atrium.

**Supplementary Figure 1. Study flow**

MRI, magnetic resonance imaging

**Supplementary Figure 2. Tricuspid Regurgitation, Left Atrial Volume Index, and Newly Diagnosed Atrial Fibrillation after Ischemic Stroke**
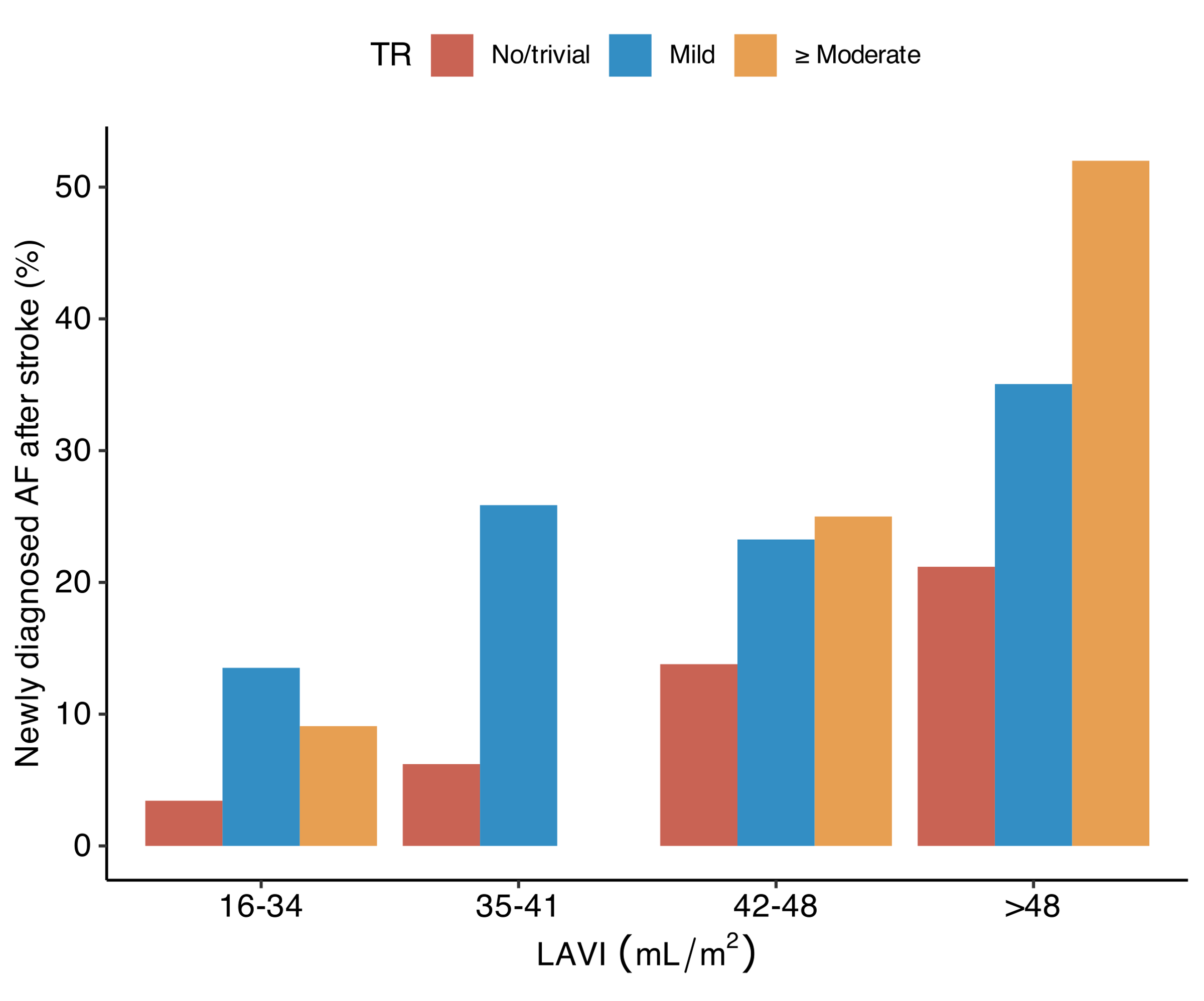


TR, tricuspid regurgitation; LA, left atrium; LAVI, left atrium volume index; AF, atrial fibrillation
